# Improvement in glycemia after glucose or insulin overload in leptin-infused rats is associated with insulin-related activation of hepatic glucose metabolism

**DOI:** 10.1186/s12986-016-0079-9

**Published:** 2016-03-01

**Authors:** Emma Burgos-Ramos, Sandra Canelles, Laura M. Frago, Julie A. Chowen, Eduardo Arilla-Ferreiro, Jesús Argente, Vicente Barrios

**Affiliations:** Department of Endocrinology, Hospital Infantil Universitario Niño Jesús, Instituto de Investigación La Princesa, Avda. Menéndez Pelayo, 65, E-28009 Madrid, Spain; Centro de Investigación Biomédica en Red de Fisiopatología de la Obesidad y Nutrición (CIBEROBN), Instituto de Salud Carlos III, Madrid, E-28009 Spain; Department of Pediatrics, Universidad Autónoma de Madrid, Madrid, E-28009 Spain; Neurobiochemistry Group, Unit of Biochemistry and Molecular Biology, Facultad de Medicina, Universidad de Alcalá, E-28871, Alcalá de Henares, Spain; Present address : IMDEA Food, CEI UAM + CSIC, Carretera de Cantoblanco 8, Madrid, E-28049 Spain

**Keywords:** Glycemia, Glycogen synthesis, Insulin signaling, Leptin, Liver, Tolerance test

## Abstract

**Background:**

Insulin regulates glucose homeostasis through direct effects on the liver, among other organs, with leptin modulating insulin’s hepatic actions. Since central leptin may modify insulin signaling in the liver, we hypothesized that leptin infusion activates hepatic glycogen synthesis following peripheral administration of a bolus of glucose or insulin, thus regulating glycemia.

**Findings:**

Oral glucose and intraperitoneal insulin tolerance tests were performed in control, intracerebroventricular leptin-treated and pair-fed rats during 14 days. An improvement in glycemia and an increase in hepatic free glucose and glycogen concentrations after glucose or insulin overload were observed in leptin-treated rats. In order to analyze whether the liver was involved in these changes, we studied activation of insulin signaling by Western blotting and multiplex bead immunoassay after leptin infusion. Our studies revealed an increase in phosphorylation of insulin receptor substrate-1 and Akt in leptin-treated rats. Examination of parameters related to glucose uptake and metabolism in the liver revealed an augment in glucose transporter 2 and a decrease in phosphoenolpyruvate carboxylase protein levels in this group.

**Conclusions:**

These results indicate that central leptin increases hepatic insulin signaling, associated with increased glycogen concentrations after glucose or insulin overload, leading to an improvement in glycemia.

## Findings

### Introduction

Leptin modulates hepatic insulin action [1] and is a key regulator of carbohydrate homeostasis. Under physiological conditions, insulin modulates glucose fluxes by suppressing the expression of gluconeogenic genes and stimulating those associated with glucose uptake. Leptin is involved in these actions through stimulation of phosphatidylinositol-3 kinase [2].

Intravenous [3] and brain infusions of leptin [4] alter hepatic glucose fluxes, improving glucose homeostasis. These effects on insulin’s actions in peripheral organs have been examined in models of obesity and diabetes [5], however; there is little information regarding the effects of an increase in central leptin bioavailability on hepatic insulin sensitivity in non-obese animals.

We have recently reported that central leptin infusion increases the hepatic response to a rise in brain insulin levels [6]. Central leptin actions affect hepatic metabolism [7, 8]; however, its actions after a rise in peripheral glucose or insulin remain only partially characterized. Thus, we hypothesized that the improvement in glycemia after oral glucose or peripheral insulin administration in chronic leptin-infused non-obese rats could be explained by changes in glucose metabolism due to leptin-related changes in hepatic insulin sensitivity.

## Methods

### Animals

Thirty-six adult male Wistar rats (250 ± 10 g) were caged with a 12-h light/dark cycle and given standard chow and water *ad libitum*. After an overnight fast, rats were anesthetized and positioned in a stereotaxic apparatus. A cannula attached to an osmotic minipump (Alzet, Durect Corporation, Cupertino, CA) containing saline (controls, C) or leptin (Preprotech, Rocky Hill, NJ, USA; 12 μg/day) was implanted and maintained during 14 days (L), as reported [6]. To discriminate the inhibitory effect of leptin on food intake, a pair-fed group (PF) was included. On the last day, twelve rats were fasted for 12 h and then sacrificed, obtaining trunk blood for the determination of glucose, leptin and insulin levels. The liver was weighed and processed for measurement of activation of insulin signaling targets, protein levels of glucose transporter (GLUT)2 and −4 and phosphoenolpyruvate carboxykinase (PEPCK). The weight of the gastrocnemius and subcutaneous and epididymal fat pads was also recorded.

Twelve rats were fasted for 12 h, followed by an oral glucose tolerance test (OGTT) (n = 4 per group). A bolus of glucose (2 g/kg body weight) was administered orally [9]. Glycemia was determined (Accu-Check Sensor) in blood samples extracted from the tail vein before glucose administration and at 15, 30, 60 and 120 min, as well as insulin levels. The liver was processed after OGTT for measurement of free glucose and glycogen concentrations.

Insulin sensitivity was assessed after fasting by performing an intraperitoneal insulin tolerance test (IPITT) [10] in the remaining twelve rats. After the injection of 1 U/kg of insulin, blood samples were drawn at 30, 60, 90 and 120 min for glucose measurements. The liver was extracted for determination of glucose and glycogen. This study was approved by the Ethics Committee of the Universidad de Alcalá de Henares.

### ELISAs

Serum leptin and insulin levels were measured using ELISA kits from Millipore Corporate Headquarters (Billerica, MA, USA). The intra- and inter-assay variations were lower than 10 %.

### Western blotting

Western blots were performed using antibodies against GLUT2, the beta chain of the insulin receptor (IRβ) and PEPCK from Santa Cruz Biotechnology (Santa Cruz, CA, USA) and anti-GLUT4 from Millipore (Temecula, CA, USA). The proteins were detected by chemiluminiscence using an ECL system. Quantification of the bands was carried-out by densitometry using a Kodak Gel Logic 1500 Image Analysis system and Molecular Imaging software 4.0 (Rochester, NY, USA). Proteins were normalized with β-actin (Thermo Scientific, Fremont, CA, USA).

### Multiplexed bead immunoassay

Phosphorylated and total protein levels of IR substrate 1 (IRS1), Akt and phosphatase and tensin homolog on chromosome 10 (PTEN) were determined by a multiplexed bead immunoassay (Millipore). A minimum of 50 beads per parameter were analyzed in the Bio-Plex suspension array system 200 (Bio-Rad). Raw data (median fluorescence intensity, MFI) were analyzed with the Bio-Plex Manager Software 4.1 (Bio-Rad Laboratories).

### Measurement of hepatic glucose and glycogen

Glucose was measured by an enzymatic method from Sigma-Aldrich (GAGO-20), in homogenized samples [11]. For quantification of glycogen, liver samples were processed as previously reported [6] and the resulting glucose concentrations determined by the same method.

### Statistical analysis

Data are expressed as mean ± SEM. Statistical analysis was carried out by one-way ANOVA or repeated measures for OGTT or IPITT followed by a Bonferroni’s test. Values were considered significantly different when the *P* value was less than 0.05. Analyses were conducted with Prisma software 4.00 (GraphPad, San Diego, CA, USA).

## Results

### General characteristics of the experimental groups

Average daily food intake was reduced in PF and L and body weight gain was lower in these groups, with a more pronounced reduction in L (Table 1). Basal values of serum glucose, leptin and insulin levels, as well as the weight of the liver, gastrocnemius and adipose tissue are given in Table 1.

**Table 1:** General characteristics of the experimental groups.

Parameter	Group		
	Control	Pair-fed	Leptin
Daily food intake (g)	10.11 ± 0.64	6.75 ± 0.40*	6.67 ± 0.39*
Δ body weight (g)	41.82 ± 3.97	22.50 ± 3.07**	3.12 ± 0.18**^##^
Glucose (mg/dl)	79.54 ± 4.62	76.70 ± 2.03	83.78 ± 3.20
Leptin (ng/ml)	4.08 ± 1.46	2.93 ± 0.38	10.62 ± 2.93**^##^
Insulin (ng/ml)	0.80 ± 0.13	0.76 ± 0.09	0.83 ± 0.32
Liver (g)	9.71 ± 0.49	10.12 ± 0.88	9.05 ± 1.04
Gastrocnemius (g)	0.96 ± 0.17	0.80 ± 0.12	1.19 ± 0.20^#^
Adipose tissue (g)	8.07 ± 0.30	5.92 ± 0.71**	3.62 ± 0.18**^##^

Values represent mean ± SEM of 4 rats per group. Weight of adipose tissue is the sum of subcutaneous and epididymal fat pads. *p<0.05, **p<0.01 vs. control group; ^#^p<0.05, ^##^p<0.01 vs. pair-fed group.

### Leptin improves glycemia after glucose or insulin administration

**Fig. 1:**
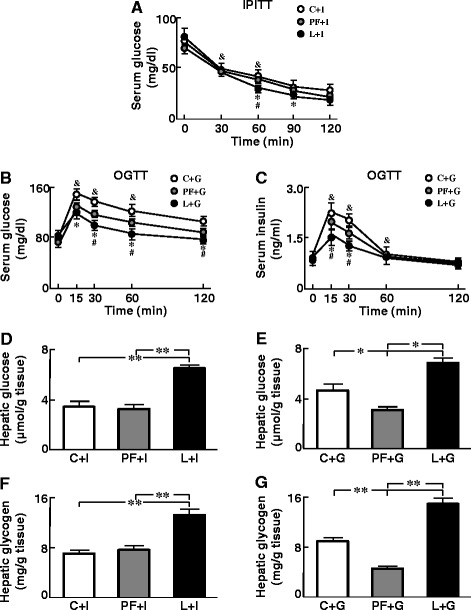
Serum parameters during OGTT or IPITT and hepatic glucose and glycogen levels after these tests. **a**. Serum glucose levels before (0 min) and during (30, 60, 90 and 120 min) an intraperitoneal (IP) insulin tolerance test (IPITT). C + I, control rats that received an IP insulin bolus; PF + I, pair-fed rats that received an IP insulin bolus; L + I, rats treated with chronic icv leptin infusion that received an IP insulin bolus. **b**. Serum glucose levels before (0 min) and during (15, 30, 60 and 120 min) an oral glucose tolerance test (OGTT). C + G, control rats that received oral glucose; PF + G, pair-fed rats that received oral glucose; L + G, rats treated with chronic icv leptin infusion that received oral glucose. **c**. Serum insulin levels before and during an OGTT. **d**. Hepatic glucose levels after an IPITT. **e**. Hepatic glucose levels after an OGTT. **f**. Hepatic glycogen concentrations after an IPITT. **g**. Hepatic glycogen concentrations after an OGTT. *p < 0.05 vs. C, ^#^ *p* < 0.05 vs PF, &*p* < 0.05 vs. previous time-point in Fig. 1a-c or *p < 0.05, **p < 0.01 vs. C or PF in Fig. 1d-g

We found no differences in basal glycemia or insulin levels. A drop in glycemia was observed throughout the IPITT in all groups, being more pronounced in L at 60 and 90 min (Fig. 1a). Administration of glucose triggered a substantial increase in glycemia (Fig. 1b) and serum insulin levels (Fig. 1c) in all groups, with this increase being lower in PF and L with respect to C and in L compared to PF.

### The effect of glucose or insulin overload on hepatic glucose and glycogen levels is potentiated by leptin

Hepatic glucose content was higher in L after IPITT (Fig. 1d) and lower in PF compared to both C and L rats and higher in L compared to C and PF rats after OGTT (Fig. 1e). Glycogen levels were higher in L after IPITT (Fig. 1f) and lower in PF compared to both Cand L rats and higher in L compared to C and PF rats after OGTT (Fig. 1g).

### Hepatic insulin signaling is activated by leptin infusion

**Fig. 2:**
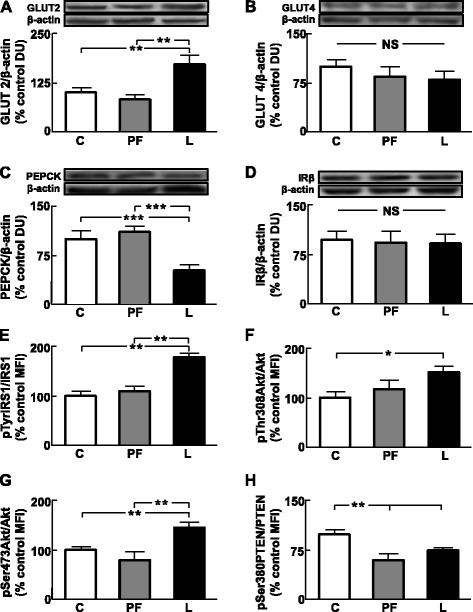
Leptin infusion modifies insulin signaling and parameters related to glucose metabolism in the liver. **a**. Relative glucose transporter (GLUT)2 protein levels. C, control rats; PF, pair-fed rats and L, rats treated with chronic icv leptin infusion. **b**. Relative GLUT4 protein levels. **c**. Relative phosphoenolpyruvate carboxykinase (PEPCK) protein levels. **d**. Relative insulin receptor beta chain (IRβ) protein levels. **e**. Relative phosphorylated (p) insulin receptor substrate (IRS)1 protein levels. **f**. Relative pAkt on threonine 308 (pThr308Akt) protein levels. **g**. Relative pAkt on serine 473 (pSer473Akt) protein levels. **h**. Relative p-phosphatase and tensin homolog on chromosome 10 (PTEN) on serine 380 (pSer380PTEN) protein levels. DU, densitometry units; MFI, median fluorescent intensity; NS, non-significant; *p < 0.05, **p < 0.01, ***p < 0.001

Hepatic GLUT2 protein levels were higher in L compared to both C and PF rats (Fig. 2a), whereas GLUT4 levels were unchanged (Fig. 2b). PEPCK protein levels were lower in L compared to the other two groups (Fig. 2c). Hepatic levels of IRβ were not modified (Fig. 2d). The phosphorylation of IRS1 was higher in L compared to C and PF rats (Fig. 2e). Phosphorylation of Akt on the Thr308 residue was increased in L with respect to C (Fig. 2f) and on Ser473 phosphorylation was increased in L compared to C and PF rats (Fig. 2g). Finally, PTEN phosphorylation was reduced in PF and L (Fig. 2h).

## Discussion

The goal of this study was to examine the effect of central leptin infusion on glycemia after a peripheral increase in glucose or insulin and the possible relationship with changes in glucose uptake and its metabolism in the liver. We found that leptin-treated rats had higher hepatic glucose and glycogen concentrations, probably due to higher levels of GLUT2 [12], thus regulating glycemia. Several differences between pair-fed and leptin-treated rats were observed, in particular, higher leptin concentrations in the leptin- infused group. In fact, leptin infusion causes hyperleptinemia [13] as intracerebroventricular leptin goes to the periphery, as previously reported [14]. In addition, the gastrocnemius of these rats weighs more than in the pair-fed group, as previously reported [15], probably related to the leptin-induced increase in carbohydrate disposal [16]. Likewise, the reduction in fat pads is most likely due to leptin’s suppression of glucose utilization [17].

Consistent with these findings, central leptin administration modifies glucose fluxes and production [18, 19] and these changes are partially mediated by increasing hepatic insulin sensitivity, as we report here. In fact, exogenous leptin has been shown to exert positive effects on peripheral insulin signaling that involve leptin-insulin cross-talk [1]. Indeed, an increase in central leptin is reported to reverse hepatic insulin resistance [7] and to correct peripheral glucose usage [20]. The insulin and leptin signaling pathways share several targets, such as Janus kinase-2, IRSs and Akt [21], and we have reported that interaction of these pathways potentiates insulin signaling [6]. While muscle most likely participates in the regulation of serum glucose levels, as leptin increases insulin sensitivity in this tissue [22], our results clearly indicate a key role of the liver in leptin’s effects on serum glucose improvement.

Tolerance tests give more accurate information than homeostasis model assessment of insulin resistance to determine insulin sensitivity [23, 24]. Here, tolerance tests reveal that the higher concentrations of glucose and glycogen in the liver of leptin-infused rats may be related with its increased insulin sensitivity. These changes seem to be due to the higher degree of phosphorylation on both the Thr308 and Ser473 residues of Akt, which is necessary to achieve full activation of the insulin signaling cascade [25].

In conclusion, our results suggest that improvement in glycemia after peripheral glucose or insulin administration in central leptin-infused rats is due, at least in part, to the previous activation of hepatic insulin signaling that may increase glucose uptake and glycogen storage, thus contributing to lower serum glucose levels.

## Abbreviations

Akt: Protein kinase B
DU: Densitometry units
GLUT: Glucose transporter
IR: Insulin receptor
IRS1: IR substrate 1
IPITT: Intraperitoneal insulin tolerance test
MFI: Median fluorescence intensity
OGTT: Oral glucose tolerance test
PEPCK: Phosphoenolpyruvate carboxykinase
PTEN: Phosphatase and tensin homolog on chromosome 10

## References

[1] Berthou F, Rouch C, Gertler A, Gerozissis K, Taouis M (2011). Chronic central leptin infusion differently modulates brain and liver insulin signaling. Mol Cell Endocrinol.

[2] Kellerer M, Koch M, Metzinger E, Mushack J, Capp E, Häring HU (1997). Leptin activates PI-3 kinase in C2C12 myotubes via janus kinase-2 (JAK-2) and insulin receptor substrate-2 (IRS-2) dependent pathways. Diabetologia.

[3] Rossetti L, Massillon D, Barzilai N, Vuguin P, Chen W, Hawkins M (1997). Short term effects of leptin on hepatic gluconeogenesis and in vivo insulin action. J Biol Chem.

[4] Cusin I, Zakrzewska KE, Boss O, Muzzin P, Giacobino JP, Ricquier D (1998). Chronic central leptin infusion enhances insulin-stimulated glucose metabolism and favors the expression of uncoupling proteins. Diabetes.

[5] Sloan C, Tuinei J, Nemetz K, Frandsen J, Soto J, Wride N (2011). Central leptin signaling is required to normalize myocardial fatty acid oxidation rates in caloric-restricted ob/ob mice. Diabetes.

[6] Burgos-Ramos E, Canelles S, Rodríguez A, Gómez-Ambrosi J, Frago LM, Chowen JA (2015). Chronic central leptin infusion modulates the glycemia response to insulin administration in male rats through regulation of hepatic glucose metabolism. Mol Cell Endocrinol.

[7] Pocai A, Morgan K, Buettner C, Gutierrez-Juarez R, Obici S, Rossetti L (2005). Central leptin acutely reverses diet-induced hepatic insulin resistance. Diabetes.

[8] Cortés VA, Cautivo KM, Rong S, Garg A, Horton JD, Agarwal AK (2014). Leptin ameliorates insulin resistance and hepatic steatosis in Agpat2−/− lipodystrophic mice independent of hepatocyte leptin receptors. J Lipid Res.

[9] Suresha RN, Ashwini V, Pragathi B, Kalabharathi HL, Satish AM, Pushpa VH (2013). The effect of carvedilol on blood glucose levels in normal albino rats. J Clin Diagn Res.

[10] Ndisang JF, Lane N, Syed N, Jadhav A (2010). Up-regulating the heme oxygenase system with hemin improves insulin sensitivity and glucose metabolism in adult spontaneously hypertensive rats. Endocrinology.

[11] Niewoehner CB, Nuttall FQ (1988). Relationship of hepatic glucose uptake to intrahepatic glucose concentration in fasted rats after glucose load. Diabetes.

[12] Sole SS, Srinivasan BP (2012). Aqueous extract of tamarind seeds selectively increases glucose transporter-2, glucose transporter-4, and islets' intracellular calcium levels and stimulates β-cell proliferation resulting in improved glucose homeostasis in rats with streptozotocin-induced diabetes mellitus. Nutr Res.

[13] Morrison CD, Daniel JA, Holmberg BJ, Djiane J, Raver N, Gertler A (2001). Central infusion of leptin into well-fed and undernourished ewe lambs: effects on feed intake and serum concentrations of growth hormone and luteinizing hormone. J Endocrinol.

[14] Maness LM, Kastin AJ, Farrell CL, Banks WA (1998). Fate of leptin after intracerebroventricular injection into the mouse brain. Endocrinology.

[15] Hamrick MW, Herberg S, Arounleut P, He HZ, Shiver A, Qi RQ (2010). The adipokine leptin increases skeletal muscle mass and significantly alters skeletal muscle miRNA expression profile in aged mice. Biochem Biophys Res Commun.

[16] Yaspelkis BB, Singh MK, Krisan AD, Collins DE, Kwong CC, Bernard JR (2004). Chronic leptin treatment enhances insulin-stimulated glucose disposal in skeletal muscle of high-fat fed rodents. Life Sci.

[17] Wang JL, Chinookoswong N, Scully S, Qi M, Shi ZQ (1999). Differential effects of leptin in regulation of tissue glucose utilization in vivo. Endocrinology.

[18] Perry RJ, Zhang XM, Zhang D, Kumashiro N, Camporez JP, Cline GW (2014). Leptin reverses diabetes by suppression of the hypothalamic-pituitary-adrenal axis. Nat Med.

[19] Kim GH, Szabo A, King EM, Ayala J, Ayala JE, Altarejos JY (2014). Leptin recruits Creb-regulated transcriptional coactivator 1 to improve hyperglycemia in insulin-deficient diabetes. Mol Metab.

[20] Cettour-Rose P, Theander-Carrillo C, Asensio C, Klein M, Visser TJ, Burger AG (2005). Hypothyroidism in rats decreases peripheral glucose utilisation, a defect partially corrected by central leptin infusion. Diabetologia.

[21] Pedersen BA, Wang W, Taylor JF, Khattab OS, Chen YH, Edwards RA, et al. Hepatic proteomic analysis revealed altered metabolic pathways in insulin resistant Akt1+/−/Akt2−/− mice. Metabolism. 2015 [doi:10.1016/j.metabol.2015.09.008]. 10.1016/j.metabol.2015.09.008 PMC4641788 26455965

[22] Roman EA, Reis D, Romanatto T, Maimoni D, Ferreira EA, Santos GA (2010). Central leptin action improves skeletal muscle AKT, AMPK, and PGC1 alpha activation by hypothalamic PI3K-dependent mechanism. Mol Cell Endocrinol.

[23] Gower BA, Alvarez JA, Bush NC, Hunter GR (2013). Insulin sensitivity affects propensity to obesity in an ethnic-specific manner: results from two controlled weight loss intervention studies. Nutr Metab (Lond).

[24] Kang ES, Yun YS, Park SW, Kim HJ, Ahn CW, Song YD (2005). Limitation of the validity of the homeostasis model assessment as an index of insulin resistance in Korea. Metabolism.

[25] Xiao L, Gong LL, Yuan D, Deng M, Zeng XM, Chen LL (2010). Protein phosphatase-1 regulates Akt1 signal transduction pathway to control gene expression, cell survival and differentiation. Cell Death Differ.

